# Dogs Discriminate Identical Twins

**DOI:** 10.1371/journal.pone.0020704

**Published:** 2011-06-15

**Authors:** Ludvík Pinc, Luděk Bartoš, Alice Reslová, Radim Kotrba

**Affiliations:** 1 Department of Animal Science and Ethology, Canine Behavior Research Center, Czech University of Life Sciences, Praha, Czech Republic; 2 Department of Ethology, Institute of Animal Science, Praha Uhříněves, Czech Republic; 3 Institute of Criminalistics, Praha, Czech Republic; 4 Department of Animal Science and Food Processing, Institute of Tropics and Subtropics, Czech University of Life Sciences, Praha, Czech Republic; Alexander Flemming Biomedical Sciences Research Center, Greece

## Abstract

Earlier studies have shown variation among experimental attempts to establish whether human monozygotic twins that are genetically identical also have identical individual scents. In none of the cases were the dogs able to distinguish all the individual scents of monozygotic twins living in the same environment if the scents were presented to them separately. Ten specially trained police German Shepherd dogs of three Czech Republic Police Regional Headquarters were used for scent identification in our study. The dogs were supposed to match scents of two monozygotic pairs (5 and 7 years old) and two dizygotic twin pairs (8 and 13 years old). Scents were collected on cotton squares stored in glass jars. Dog handlers were blind to the experiment details. In each trial (line-up), one scent was used as a starting scent and the dog was then sent to determine if any of the 7 presented glass jars contained a matching scent. Scents of children of similar ages were used as distractors. In the matching procedure, the dogs matched correctly the scent of one twin with the other, as well as two scents collected from every single identical and non-identical twin to prove their efficacy and likewise, the presence of the matching twin scent in any given glass jar. All dogs in all trials distinguished correctly the scents of identical as well as non-identical twins. All dogs similarly matched positively two scents collected from the same individuals. Our findings indicated that specially trained German Shepherd dogs are able to distinguish individual scents of identical twins despite the fact that they live in the same environment, eat the same food and even if the scents are not presented to them simultaneously.

## Introduction

Scent identification line-up performed by trained dogs is a method used by European countries such as the Czech Republic [1], [2], Poland [3], Russia [4], Hungary [5], Denmark, and Netherland [6], [7] However, it has not gained widespread acceptance in the United States, mainly due to the lack of scientific studies demonstrating the reliability of this method [8]. The line-ups are performed in accordance with different training principles and forensic and law regulations. Essentially, a human scent left by a perpetrator at a crime scene is later matched with the scent sample taken from the detained suspect [9]. Numerous studies have shown that the major histocompatibility complex (MHC) plays a decisive role in olfactory individual recognition, olfactory kin recognition as well as in reproductive behaviors in mice and other mammals [10]–[12]. Apparently MHC may play a role even in human scent attractiveness [13], [14]. As monozygotic twins are supposed to have identical MHC genes, it is not surprising that dogs are not able to reliably distinguish one identical twin from the other. Kalmus [15] was the first who tested the ability of dogs to discriminate identical twins scents. He used nine dogs that varied greatly “in intelligence, perseverance and the degree to which they had been trained”. The scent donors were 17 men, 9 women and 5 children. Some individual dogs that he had at his disposal, performed retrieving and tracking experiments to show their ability to distinguish between identical twins. While in the retrieving experiments, the dogs did not seem to perceive any difference, in the tracking experiments dogs could distinguish the body scents of identical twins provided the scents were offered simultaneously. However, when the scent of one twin was offered in place of the scent of the other, and in the latter's absence, it was picked out from the scents of other people. The author thus suggested that although identical twins have more similar body scents than those of any other people, their individual body scents could be distinguished by well trained dogs. Hepper [16] used a set of identical and nonidentical twins as scent donors tested by four dogs of two breeds. He concluded that, “Twins are discriminable by dogs if they differ genetically, or, if identical, they are subject to differences in their environment, particularly diet. However, if they are both genetically identical and fed the same diet then, to dogs at least, they do not produce sufficiently different scents to make them discriminable”. To distinguish identical twins, Sommerville et al. [17] used a German shepherd dog to match their scents. The only dog used, made 13 correct matches out of 17 for unrelated people. On the identical twins it matched correctly 14 out of 21 trials that is a result equal to the random score. When the dog was presented with the sweat fraction that seemed to display individual differences, it matched correctly samples of unrelated people in 11 out of 14 cases. The samples of identical twins were retrieved indiscriminately. More recently, Harvey et al. [18] used bloodhounds trained in human scent discrimination to differentiate between monozygotic twins living together and apart, related and nonrelated persons who were also living together and apart. The dogs were used in two different testing scenarios. In the first one, the dogs were presented with a scent sample and were supposed to not follow a track laid down by another person. In the second test, the dogs were required to match a scent sample with one of two tracks: correct and incorrect. In this case, the track layers walked side by side and then split in a “Y” shaped pattern. In the first test, none of the 13 dogs performed better than chance on the 9 pairs of monozygotic twins living together and only 3 dogs performed better than chance on the same number of monozygotic twins living apart. In the second test only 1 dog out of 9 performed better than chance on the tracks laid by 9 monozygotic twins living together and only 5 dogs performed better than chance on the tracks laid by 9 monozygotic twins living apart. Kalmus [15], Hepper [16], and Harvey et al. [18] in the previous studies accordingly concluded that people have individual odorotypes as a result of their genomes. They also showed that at least some individual dogs were able to differentiate monozygotic twins [15], [18]. It suggested that there might be individual odor types acquired over the ontogeny of a person independent of their genetics. The apparent inconsistency in the results of the previous studies may be attributed to the different level of training of the dogs used in the respective experiments. Therefore, we hypothesized that police dogs with the highest level of training would be able to distinguish individual scents of monozygotic twins even if they lived together and ate the same food, regardless of any scent differences acquired over their ontogeny. A dog should also be more successful in differentiating smells with the increasing age of identical twins.

## Results

Each of the ten dogs performed 12 matching procedures (60 line-ups) as described in Table 1. All dogs invariably discriminated correctly the scents of all monozygotic and dizygotic twins and also made a positive match on two scents collected from the same individuals. (The Sign test, correct results n = 120, incorrect results n = 0, P<0.001).

**Table 1:** Scent identification matching procedures performed by one dog.

Dog ID	Type of twins	Matching procedure (in random order)	Number of line-ups	Correct results (n)	Incorrect results (n)
Gabi	Monozygotic twins	1A×1A	5	1	0
		1A×1B	5	1	0
		1B×1B	5	1	0
		2A×2A	5	1	0
		2A×2B	5	1	0
		2B×2B	5	1	0
	Dizygotic twins	3A×3A	5	1	0
		3A×3B	5	1	0
		3B×3B	5	1	0
		4A×4A	5	1	0
		4A×4B	5	1	0
		4B×4B	5	1	0
	Total:		60	12	0

As all dogs used in the experiment performed in exactly the same way, with no incorrect alerts, performance of other dogs are not shown.

## Discussion

The findings of this study are in accord with the hypothesis that properly trained dogs are able to discriminate correctly individual scents of monozygotic twins even if they live in the same household and eat the same food. Unlike other studies [15], dogs used in our research were trained police dogs, certified and routinely used exclusively for scent identification line-ups. Some dogs used by Kalmus [15] and Harvey et al. [18] were also trained police canines but not exclusively for scent identification line-ups. Moreover, the level of efficacy used in those earlier studies was not uniform. In the case of the study conducted by Kalmus [15], only those that performed best were used. Another point to consider is that Hepper [16] did not offer any information on the training of the 4 dogs used in his study. Likewise, Sommerville et al. [17] used only one dog in their study without a description of the dog's training. The results thus suggest in accordance with [15] that the level of training may be critical in these kinds of experiments. It seems that by the age of 5 years, specific individual scents of identical twins are recognizable by specially trained German Shepherd dogs. Further research should be focused on the age of the identical twins and whether or when their scents start to differentiate.

## Materials and Methods

**Figure 1:**
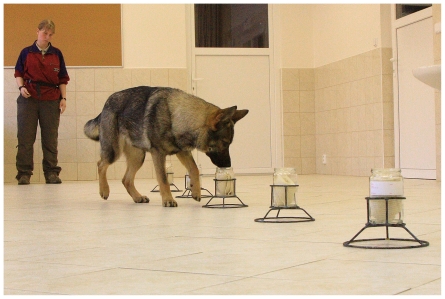
A photograph of the dog sniffing the scent sample in a line-up.

**Figure 2:**
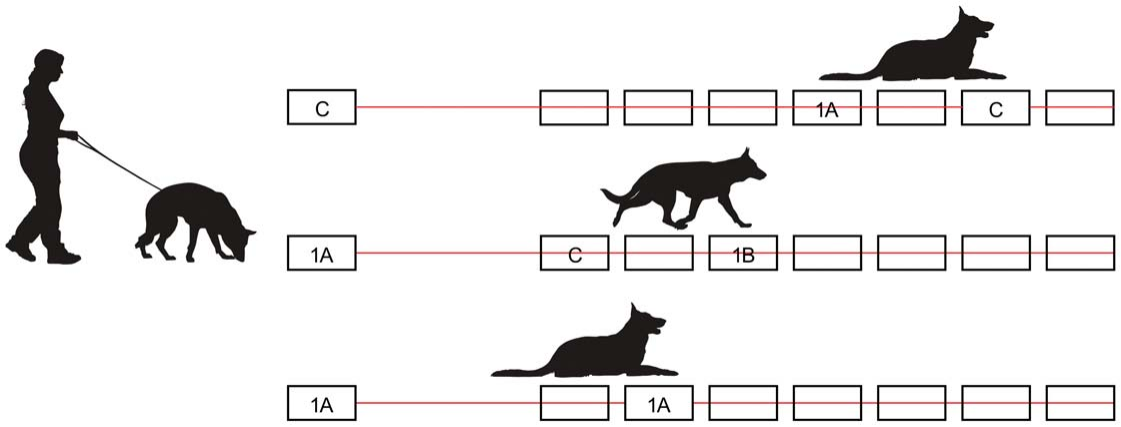
Scheme showing examples of how the scent identification line-ups were performed. **1A** is a scent of the first twin and **1B** is a scent of the second twin of the same pair. The blank rectangles stand for the distracting scents. An interrupted line signifies that the dog alerted to the scent. **C** is a control scent or training scent used by the handler for training purposes to let the dog make positive matches. The dogs moved from left to right. The separated rectangles on the left represent starting scent. In the first line-up, a twin's scent served as an attractor in a control line-up. Any given dog smelled the starting scent and was sent to search for the matching scent, while ignoring the attractor (top). A correct result of the middle line-up would be “not matched” i.e. the dog did not match the two scents. The bottom row shows matching of two scents collected from a single twin. In this case the correct result would be “matched” as required.

Scent identification in the Czech Republic is done by specially trained canine teams assigned to the canine units that are a part of regional headquarters of the Czech Republic Police. To become a scent identification canine officer the applicant has to have at least 3 years of practice as a regular patrolling canine handler. Then the chosen handlers go through a 3-month special scent identification course at the Police Canine Training Center. The scent identification is provided in accordance with the Code of Criminal Procedures no. 141/1961 Sb. During the scent identification, the trained dog sniffs at the starting scent (scent sample collected at the crime scene or from the suspect) also called “smeller” [19] and then searches in the line-up of odors (Fig. 1) for a matching odor (target scent). The target scents in this study were collected from two sets of monozygotic twins, aged 5 (boys) and 7 (girls), and two sets of dizygotic twins, aged 8 (girls) and 13 (boys). For all the children participating in the study a written informed consent was obtained from their parents. The parents were always present in the next room when the scent samples and in the same room when buccal swabs were collected. Each pair of twins lived in the same environments and ate the same food. Monozygozity was verified by DNA typing performed by the Department of Genetics in the Institute of Criminalistics Prague. Samples for DNA typing were collected in the form of buccal swabs from both pairs of monozygotic twins (separate samples from one pair of twins were labeled “1A” and “1B”, samples from the second pair of twins were labeled “2A” and “2B”). All samples were processed using standard methods and instruments that are used in routine forensic casework samples and/or database samples analysis. DNA was extracted using QIAamp® 96 DNA Swab BioRobot® Kit, with BioRobot Universal System (QIAGENE). Sixteen DNA polymorphic loci (fifteen STR loci and Amelogenin) were then amplified using PowerPlex® 16 System (Promega) in accordance with the manufacturer's instructions. The amplified products were separated and detected using the Applied Biosystems 3130xl Genetic Analyzer using reference sequenced ladders. Laboratory internal control standards and kit controls were used to ensure that reliable results could be obtained. Final data analyzing was performed using the Applied Biosystems GeneMapper® ID-X Software Version 1.1. As expected, two male DNA profiles identical in all defined polymorphic loci were obtained from samples “1A” and “1B”, and two female DNA profiles identical in all defined polymorphic loci were obtained from samples “2A” and “2B”. The results of the DNA typing confirmed that both individuals in each pair of twins have identical genetic information and therefore they have the same DNA profile. The forensic DNA typing methods currently in use are not able to distinguish one monozygotic twin from the other. Distractor samples were collected from 5 boys aged between 6 and 7 years old and from 5 girls aged from 6 to 7 years old. Any contact between these participants was prevented. The distractors were used as non-target scents in the line-ups. Ten German Shepherds (7 females, 3 males) handled by 5 canine officers (each handling two dogs) used in the experiment were scent identification police canines used by the Czech Republic Police that served with their handlers at the regional police headquarters in the cities of Brno, Hradec Králové and Plzeň. The canines had routinely performed scent identification line–ups as a part of criminal investigation procedures before participating in the experiment. Scent samples were collected and stored according with the protocol routinely used by the crime scene technicians when collecting scent samples from suspects. Sterile cotton absorbent squares (ARATEX™) size 30×30 cm were stored in a glass jar with twist off lid, labeled and sealed in evidence plastic bags. Before the scent collection, the twins were separated into different rooms with no apparent differences in background odors. Both rooms were connected by an open door allowing air flow prior to the scent collection. Also distracting scents were collected in the same rooms. As the scent identification canines had been trained and routinely used to search for matching human scents regardless of background odors, and not to differentiate the scents, the background odors were not supposed to play a role. All experimental subjects opened the glass jar and applied the ARATEX™ cotton square themselves by putting it on the naked skin in the belly region for 20 minutes. During the scent absorption the children were asked to put the lid back on the glass jar. Next, they put the square with the scent back into the glass jar and an adult assistant tightened the lid. The assistant was always the same person (LP). Two scent samples were collected from each twin. The glass jars were then transported to the Czech University of Life Sciences Praha, where they were stored in room temperature (i.e. in similar conditions as scent samples are stored at the police canine facilities). The actual scent identification was conducted at the police scent identification canine facilities. Handlers were blind to the position of the target scents in the line-up and expected results. They were given the glass jars with the scent samples and asked to do the scent identification procedure and write down an official report detailing the outcome of the scent identification task, just as in an actual criminal investigation. Each scent matching procedure comprised five line-ups and resulted in a report stating that two presented scent samples matched or not. Two of the line-ups were of the type shown in Fig. 2 (top). The other three line-ups were a random mixture of the types in Fig. 2 (middle and bottom). Prior to the line-ups, the lids of the glass jars had been removed and the glass jars containing the ARATEX™ squares arranged in lines of seven glass jars in total. All handlers were using control scents during the matching procedure. The controls were scent samples collected from particular persons with no links to other experimental subjects. Controls served a double function: as an attractivity test and to check the general working ability and accuracy of the dogs. Seven glass jars were repeatedly present, containing one twin scent, one control scent, and five scents used as distractors. All scents were left in the line-up until the scent identification procedure was finished. The glass jars were rearranged after each line-up. As described in Fig. 2 (top), in the first line-up, one of the target twin's scent served as a distractor in a controlled line-up. Any given dog smelled the starting scent and was sent to search for the matching scent, while ignoring the attractor, and then alerting at the control scent, which was always placed in line behind the attractor (Fig. 2). This assured that the scent itself was not attractive to the dog. Then the handler opened the glass jar containing a scent of the other twin of the same pair and used it as a starting scent. The control scent previously used was left in the line-up (Fig. 2 middle). During the next phase, the dog again was sent to match the control scents while the twin scent was left in the line. The positions of the twin and control scents, as well as, the distractors were methodologically changed after each line-up. The procedure was repeated once again, until the matching procedure of 5 line-ups was completed. The same protocol was used when the dogs were performing the matching test of two scents collected from the same twin. Two scent samples collected from each twin were matched to show that there was a scent present in each glass jar and that the dogs could perceive and signal its presence (Fig. 1 bottom). All dogs signaled the presence of target scents by lying down.
